# Supplementary material for: Lsm7 phase-separated condensates trigger stress granule formation
Source: Nat Commun. 2022 Jun 28;13:3701. doi: 10.1038/s41467-022-31282-8 (PMC9240020; doi:10.1038/s41467-022-31282-8)
Supplement: Supplementary file 1 — Supplementary Information [file 41467_2022_31282_MOESM1_ESM.pdf]

Supplementary Information for

Lsm7 phase-separated condensates trigger stress granule formation

Michelle Lindström[1†], Lihua Chen[1,2*†], Shan Jiang[1], Dan Zhang[1], Yuan Gao[1], Ju Zheng[3], Xinxin Hao[1], Xiaoxue Yang[1], Arpitha Kabbinale[1], Johannes Thoma[1,4], Lisa C. Metzger[1,4], Deyuan Y. Zhang[5], Xuefeng Zhu[6], Huisheng Liu[2], Claes M. Gustafsson[6], Björn M. Burmann[1,4], Joris Winderickx[3], Per Sunnerhagen[1] & Beidong Liu[1*]

[†] These authors contributed equally: Michelle Lindström, Lihua Chen
* These authors jointly supervised this work: Beidong Liu, beidong.liu@cmb.gu.se; Lihua Chen, chen_lihua@gzlab.ac.cn

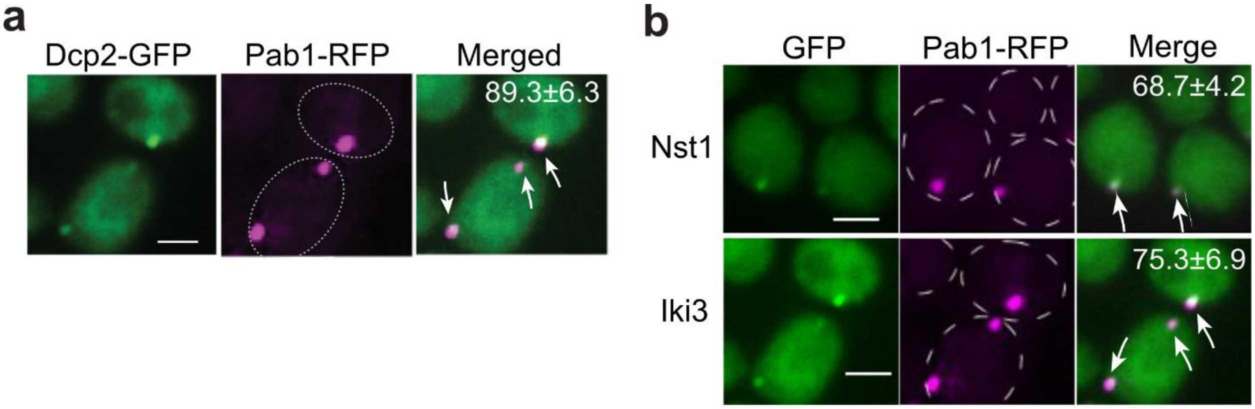

Supplementary Fig. 1. Dcp2, Nst1 and Iki3 co-localize with Pab1 under 2-DG treatment.

a, Fluorescence microscopy of Dcp2-GFP and Pab1-RFP granule components. Scale bar indicates 2 μm. White arrows indicate co-localizing GFP foci and RFP granules. Numbers in the upper right-hand corner indicate the fraction (%) of GFP foci that co-localize with RFP granules based on data from four biologically independent experiments (mean ± S.D). At least 200 cells were analyzed for each experiment. **b**, Fluorescence microscopy of granule components of Nst1 or Iki3 tagged with GFP. Stress granule marker Pab1 is tagged with RFP. Scale bar indicates 2 μm. White arrows indicate co-localizing GFP foci and RFP granules. Numbers in the upper right-hand corner indicate the fraction (%) of GFP foci that co-localize with RFP granules based on data from four biologically independent experiments (mean ± S.D). At least 200 cells were analyzed for each experiment. Source data are provided as a Source Data file.

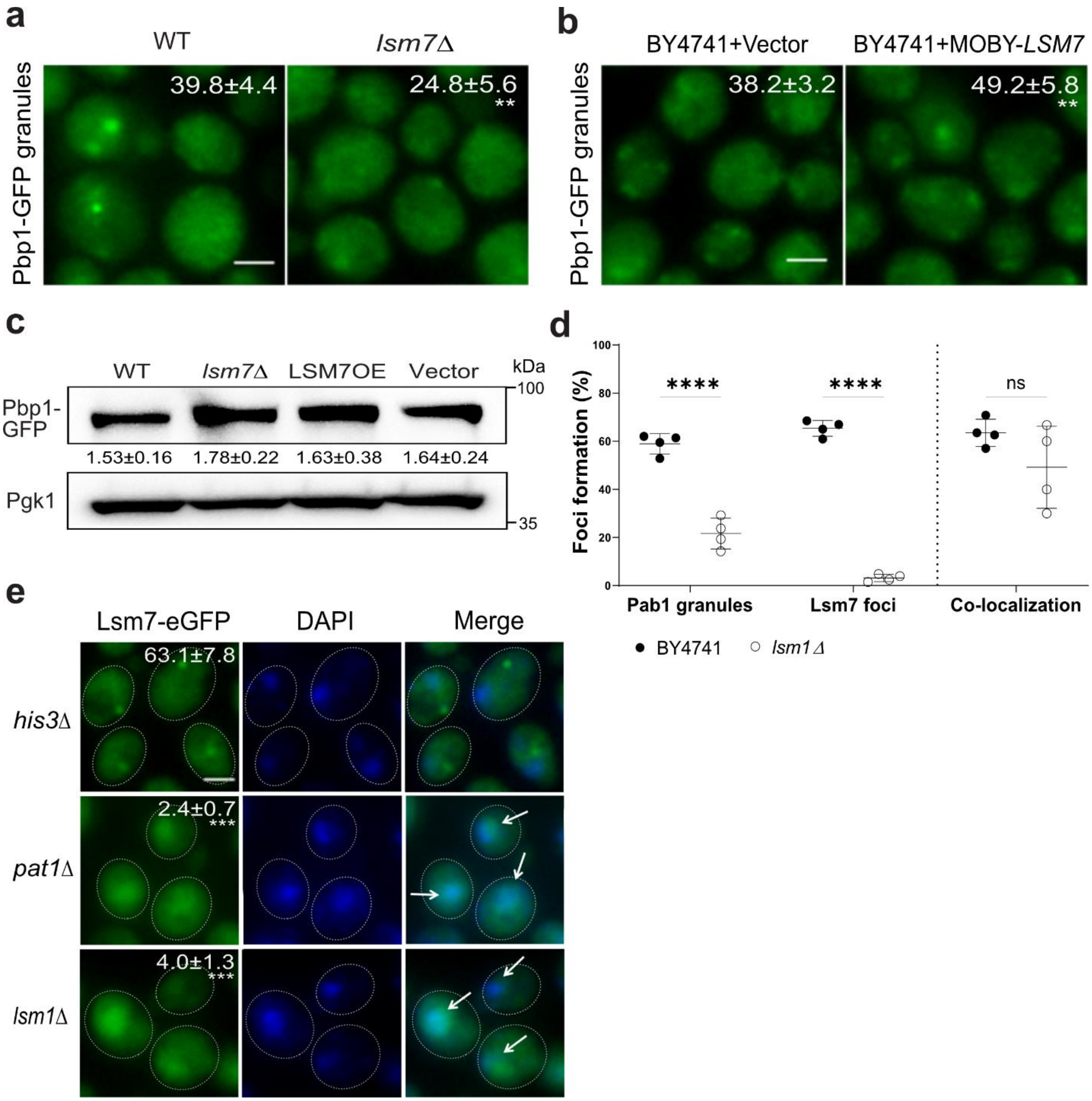

Supplementary Fig. 2. The Lsm7 effects on SGs are not specific to Pab1.
When applicable scale bar indicates 2 µm and stress was induced by 2h 2-DG treatment. Values represent percentage of foci formation or co-localization and are shown as mean ± S.D. **a,** Deletion of *LSM7* decreases Pbp1-GFP granule formation as compared to the WT control (BY4741). Three (WT) and six (*lsm7Δ*) biologically independent experiments were examined and >200 cells were analyzed for each (unpaired two-tailed t-test with Welch's correction). ** p = 0.0062. **b**, Overexpression of *LSM7* increases Pbp1-GFP granule formation, as compared to empty vector control. Six (Vector) and five (Moby-*LSM7*) biologically independent experiments were examined and >200 cells were analyzed for each (unpaired two-tailed t-test with Welch's correction). ** p = 0.0091. **c**, The Ppb1-GFP protein expression levels were not changed in the *lsm7*Δ or the *LSM7* overexpression strain. Pbp1-GFP expression levels in the WT (BY4741), *lsm7*Δ, and *LSM7*/empty

vector overexpression strains were determined by Western blotting. Data are representative of three independent experiments. Values are means ± S.D of the arbitrary units (intensity of target bands normalized to Pgk1 levels) for each clone. **d**, Deletion of *LSM1* results in decreased SG and Lsm7 foci formation. Four biologically independent experiments were examined and >200 cells were analyzed for each (two-way ANOVA followed by Šídák's test). **** $p<0.0001$, ns = 0.0661. **e**, Deletion of *PAT1* or *LSM1* results in a reduced number of Lsm7-eGFP foci, and a predominantly nuclear localization of Lsm7. Arrows indicate overlapping eGFP and DAPI signal. Four (*his3*Δ, *lsm1*Δ) and three (*pat1*Δ) biologically independent experiments were examined and >200 cells were analyzed for each (unpaired two-tailed t-test with Welch's correction). *** $p = 0.0005$. Source data are provided as a Source Data file.

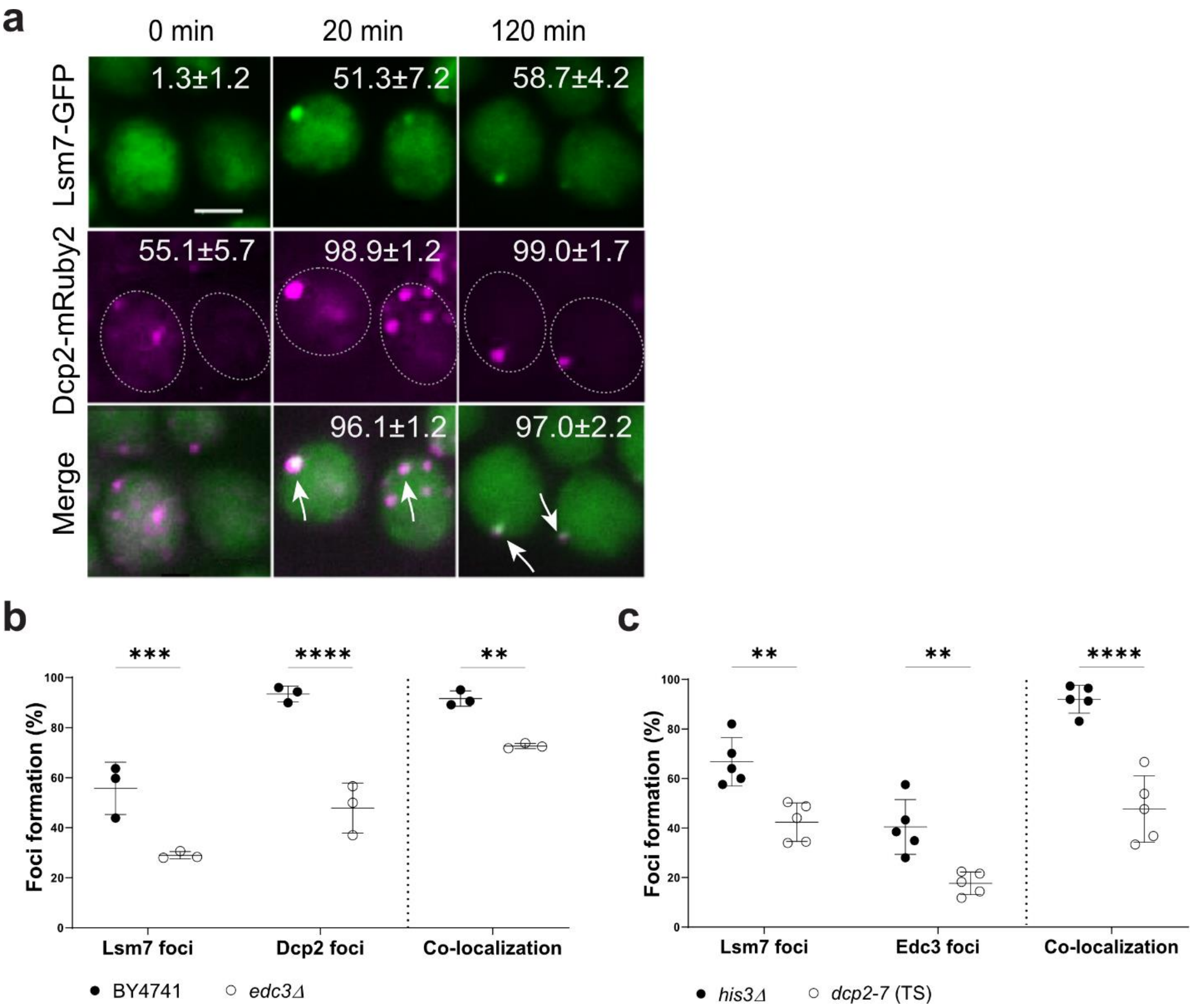

Supplementary Fig. 3. Defective PBs decrease Lsm7 foci formation.

a, Lsm7 does not form foci under unstressed conditions Like Dcp2. 0 min pre-treatment; 20 min and 120 min of 2-DG stress. Values represent percentage of cells with Lsm7-GFP or Dcp2-mRuby2 foci and are shown as means ± S.D. The bottom horizontal panel displays co-localization ratios (arrows). Four biologically independent experiments were examined and >200 cells were analyzed for each. Scale bar indicates 2 µm. **b**, Deletion of *EDC3* results in decreased PB and Lsm7 foci formation. Individual value points are shown with mean ± S.D for percentage of cells with Lsm7-mRuby2 and Dcp2-GFP foci in the WT (BY4741) and *edc3Δ* mutant, as well as the co-localization percentage. Three biologically independent experiments were examined and >200 cells were analyzed for each (two-way ANOVA followed by Šídák's test). ** p = 0.0084; *** p = 0.0006; **** p<0.0001. **c**, PB and Lsm7 foci formation is reduced in the temperature sensitive *dcp2-7* allele. Strains were grown at non-permissive temperature 37°C. Individual value points are shown with mean ± S.D for percentage of cells with Lsm7-eGFP and Edc3-RFP foci in the control (*his3Δ*) and *dcp2-7* mutant, as well as the co-localization percentage. Five biologically independent experiments were examined and >200 cells were analyzed for each (two-way ANOVA followed by Šídák's test). From left to right: ** p = 0.001; ** p<0.0021, **** p<0.0001. Source data are provided as a Source Data file.

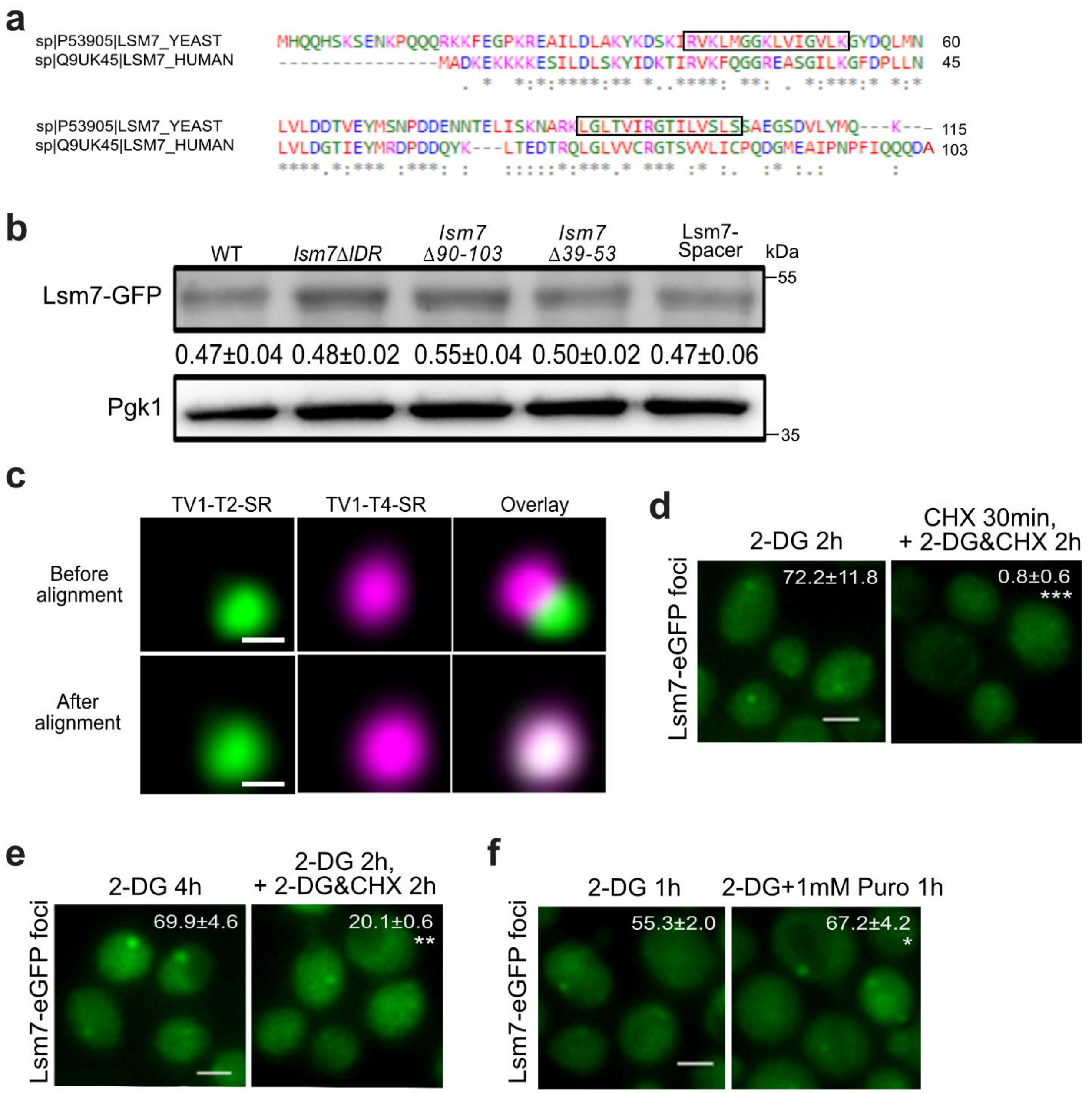

Supplementary Fig. 4. Lsm7 foci formation regulates SG induction

If not stated otherwise, scale bar indicates 2 µm. **a**, Yeast Lsm7 and human Lsm7 protein sequence alignment by using CLUSTALO program shows 36.364 % sequence identity. The sequences in black boxes are hydrophobic regions predicted by using three online tools (see Methods). **b**, Deletion of phase separation predicted domains or addition of spacer domain does not affect Lsm7-GFP protein expression, as compared to the WT (BY4741). Data are representative of three biologically independent experiments. Values are mean ± S.D of the arbitrary units (intensity of target bands normalized to Pgk1 levels) for each clone. **c**, Alignment for 3D-SIM to study the co-localization of Lsm7eGFP foci and Pab1 RFP granules. The ELYRA PS.1 LSM780 setup from Zeiss (Carl Zeiss, Jena Germany) was used and corrected for chromatic aberration in x-, y-, and z-directions using multicolor beads. All obtained images were examined and aligned accordingly.

The transformations were then applied to align the two-channel images (TV1-T2-SR and TV1-T4-SR). Scale bar indicates 0.2 µm. **d**, Cycloheximide treatment blocks Lsm7 foci formation. Cells were pre-treated with cycloheximide (30 min) prior to addition of 2-DG for 2h. Values represent percentage of cells with Lsm7-eGFP foci and are shown as mean ± S.D. Five (2-DG 2h) and four (pre-treatment) biologically independent experiments were examined and >200 cells were analyzed for each (unpaired two-tailed t-test with Welch's correction). *** p = 0.0002. **e**, Cycloheximide treatment can disassemble pre-formed Lsm7 foci. Cells were treated with 2-DG for 2h prior to addition of cycloheximide for 2 more hours. Values represent percentage of cells with Lsm7-eGFP foci and are shown as mean ± S.D. Three biologically independent experiments were examined and >200 cells were analyzed for each (unpaired two-tailed t-test). ** p = 0.0024. **f**, Puromycin enhances the 2-DG induced Lsm7 foci formation. Cells were treated with 2-DG and puromycin for 1 h. Values represent percentage of cells with Lsm7-eGFP foci and are shown as mean ± S.D. Three biologically independent experiments were examined and >200 cells were analyzed for each (unpaired two-tailed t-test). * p = 0.0232. Source data are provided as a Source Data file.

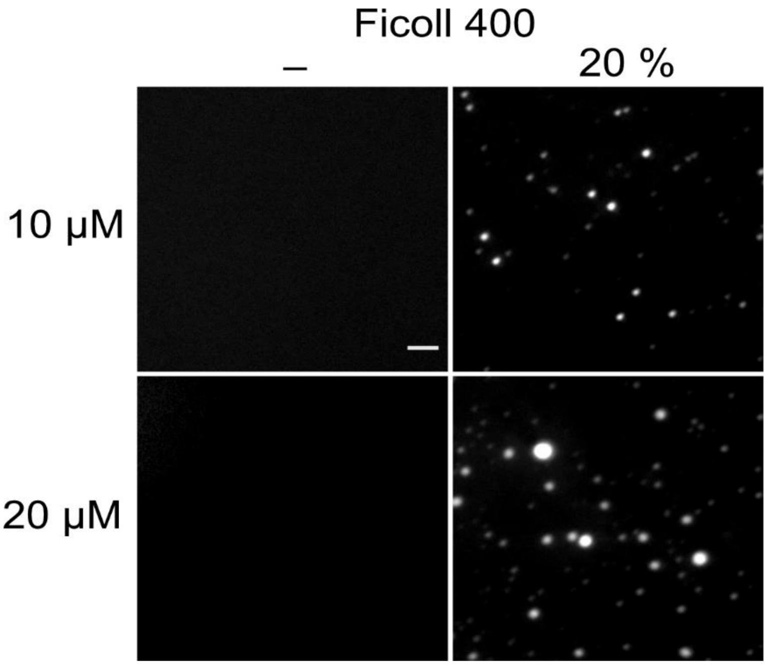

Supplementary Fig. 5. Lsm7-GFP protein undergoes phase separation *in vitro* in the presence of 20 % Ficoll 400. Lsm7-GFP was analyzed for the formation of condensates at room temperature with or without the addition of 20 % Ficoll 400 (pH 7.4, NaCl 150 mM). Images show representative data from three independent experiments. Scale bar, 2 µm.
