# Supplementary material for: Lsm7 phase-separated condensates trigger stress granule formation
Source: Nat Commun. 2022 Jun 28;13:3701. doi: 10.1038/s41467-022-31282-8 (PMC9240020; doi:10.1038/s41467-022-31282-8)
Supplement: Supplementary file 3 — Description of Additional Supplementary Files [file 41467_2022_31282_MOESM3_ESM.pdf]

File name: Supplementary Data 1

Description: Strains and plasmids used in this study.

File name: Supplementary Data 2

Description: Primers used for construction of mutants and fluorescently-tagged strains.

File name: Supplementary Data 3

Description: Complete hit list of SG components screen.

File name: Supplementary Movie 1

Description: 3D-SIM surface reconstruction of Lsm7 and Pab1 foci.
