# Supplementary material for: Lsm7 phase-separated condensates trigger stress granule formation
Source: Nat Commun. 2022 Jun 28;13:3701. doi: 10.1038/s41467-022-31282-8 (PMC9240020; doi:10.1038/s41467-022-31282-8)
Supplement: Supplementary file 8 — Reporting Summary [file 41467_2022_31282_MOESM8_ESM.pdf]

nature portfolio

Corresponding author(s): Beidong Liu
Lihua Chen

Last updated by author(s): May 3, 2022

Reporting Summary

Nature Portfolio wishes to improve the reproducibility of the work that we publish. This form provides structure for consistency and transparency in reporting. For further information on Nature Portfolio policies, see our Editorial Policies and the Editorial Policy Checklist.

Statistics

For all statistical analyses, confirm that the following items are present in the figure legend, table legend, main text, or Methods section.

n/a	Confirmed	
☐	☒	The exact sample size (*n*) for each experimental group/condition, given as a discrete number and unit of measurement
☐	☒	A statement on whether measurements were taken from distinct samples or whether the same sample was measured repeatedly
☐	☒	The statistical test(s) used AND whether they are one- or two-sided<br>*Only common tests should be described solely by name; describe more complex techniques in the Methods section.*
☒	☐	A description of all covariates tested
☐	☒	A description of any assumptions or corrections, such as tests of normality and adjustment for multiple comparisons
☐	☒	A full description of the statistical parameters including central tendency (e.g. means) or other basic estimates (e.g. regression coefficient) AND variation (e.g. standard deviation) or associated estimates of uncertainty (e.g. confidence intervals)
☐	☒	For null hypothesis testing, the test statistic (e.g. *F*, *t*, *r*) with confidence intervals, effect sizes, degrees of freedom and *P* value noted<br>*Give P values as exact values whenever suitable.*
☒	☐	For Bayesian analysis, information on the choice of priors and Markov chain Monte Carlo settings
☒	☐	For hierarchical and complex designs, identification of the appropriate level for tests and full reporting of outcomes
☒	☐	Estimates of effect sizes (e.g. Cohen's *d*, Pearson's *r*), indicating how they were calculated

Our web collection on statistics for biologists contains articles on many of the points above.

Software and code

Policy information about availability of computer code

Data collection	For the high-throughput imaging, MetaXpress (Version 3.1) software was used to obtain fluorescent sample images. For 3D-SIM acquisition, super-resolution processing and calculation as well as for 3D reconstruction, the Zen2012 software (Carl Zeiss, Jena Germany) and Imaris 7.2.3 were used. For FRAP, ZEN 2.3 software was used.
Data analysis	MetaXpress (Version 3.1) software was used to manually analyze the screen images and any co-localization. ImageJ 1.53c was used to visualize and quantify fluorescent images. GraphPad Prism version 9 (Graphpad, Inc.) was used to perform statistics and prepare graphs.<br>Relative protein expression was analyzed by the Odyssey® imaging system (Licor) or Bio-Rad ChemiDoc MP Imaging System.<br>Yeast and human protein sequence alignment was done by using CLUSTALO program (1.2.2).<br>Protein interaction analysis was performed by using Osprey 1.2.0.

For manuscripts utilizing custom algorithms or software that are central to the research but not yet described in published literature, software must be made available to editors and reviewers. We strongly encourage code deposition in a community repository (e.g. GitHub). See the Nature Portfolio guidelines for submitting code & software for further information.

Data

Policy information about availability of data

All manuscripts must include a data availability statement. This statement should provide the following information, where applicable:

- Accession codes, unique identifiers, or web links for publicly available datasets
- A description of any restrictions on data availability
- For clinical datasets or third party data, please ensure that the statement adheres to our policy

The authors declare that all data supporting the findings of this study are available within the paper and/or the supplementary information files and source data. Any domain predictions and interaction analysis were performed by using the following openly available databases:

Intrinsically disordered regions and disordered binding regions were predicated by the IUPred2 database (https://iupred2a.elte.hu/).

Prediction of prion-like domains was done with the PLAAC database (http://plaac.wi.mit.edu/).

The prediction of LLPS propensity in yeast was obtained by using the catGRANULE database (http://www.tartaglialab.com/).

Hydrophobicity prediction was done with the ExPASy - ProtScale database (Kyte & Doolittle, https://web.expasy.org/protscale/).

Lsm7 structural predictions and sequence propensities were performed with FELLS database (http://old.protein.bio.unipd.it/fells/entry/LBD0KzUVuBCAGeCVPV5bSS8Hodg?name=Lsm7&session=5f5b7d1832d0d67607ccd8de).

Prediction of Lsm7 "hot spots" of aggregation was performed with AGGRESCAN database (http://bioinf.uab.es/aggrescan/).

Physical interactions between proteins were added according to the BioGRID interaction database (https://thebiogrid.org/).

Field-specific reporting

Please select the one below that is the best fit for your research. If you are not sure, read the appropriate sections before making your selection.

☒ Life sciences ☐ Behavioural & social sciences ☐ Ecological, evolutionary & environmental sciences

For a reference copy of the document with all sections, see nature.com/documents/nr-reporting-summary-flat.pdf

Life sciences study design

All studies must disclose on these points even when the disclosure is negative.

Sample size	No statistical methods were used to predetermine sample size, but were chosen according to the standards for molecular and phenotypic characterization of in vivo and in vitro cellular models.
Data exclusions	No data were excluded from the analyses.
Replication	At least three biological replicates per strain were included per assay. All attempts at replication were successful.
Randomization	When analyzing fluorescent cell images, random imaging sites were selected. Samples were allocated into experimental groups randomly.
Blinding	The co-localization screen was performed and analyzed blinded. For individual hits confirmation and mutant analysis, images were taken randomly (blinded) to avoid any bias on sampling. The investigators were blinded to group allocation during data collection and analysis.

Reporting for specific materials, systems and methods

We require information from authors about some types of materials, experimental systems and methods used in many studies. Here, indicate whether each material, system or method listed is relevant to your study. If you are not sure if a list item applies to your research, read the appropriate section before selecting a response.

Materials & experimental systems

n/a	Involved in the study
☐	☒ Antibodies
☒	☐ Eukaryotic cell lines
☒	☐ Palaeontology and archaeology
☒	☐ Animals and other organisms
☒	☐ Human research participants
☒	☐ Clinical data
☒	☐ Dual use research of concern

Methods

n/a	Involved in the study
☒	☐ ChIP-seq
☒	☐ Flow cytometry
☒	☐ MRI-based neuroimaging

Antibodies

Antibodies used

Primary: mouse anti-Pgk1 (Invitrogen, Cat# 459250, monoclonal (22C5D8), 1:1000), rabbit anti-GFP (Abcam, Cat# ab6556, 1:5000; Abcam, Cat# ab290, 1:10000), rabbit anti-RFP (Abcam, Cat# ab62341, 1:2000), mouse anti-GFP (Santa Cruz, Cat# sc-9996, monoclonal (B-2), 1:200), mouse anti-Pab1p (EnCor Biotechnology, Cat# MCA-1G1, monoclonal (1G1), 1:100), rabbit anti-FLAG (Sigma-Aldrich, Cat# F7425, 1:100).

Secondary: HRP conjugated goat anti-mouse IgG (H+L) (Invitrogen, Cat# 62-6520, 1:5000 (Fig. 1e and 2c), 1:3000 (Supplementary Fig.2c and 4b), goat anti-rabbit DyLight 650 (Invitrogen, Cat# 84546, 1:5000).

Validation

Mouse anti-Pgk1 (Invitrogen, #459250): Manufacturer validated in Saccharomyces cerevisiae (PGK1) for WB and in numerous publications.

Rabbit anti-GFP (Abcam, ab6556): Manufacturer validated in any species (species independent) for WB and in numerous publications.

Rabbit anti-GFP (Abcam, ab290): Manufacturer validated in any species (species independent) for WB and in numerous publications.

Rabbit anti-RFP (Abcam, ab62341): Manufacturer validated for recombinant fragment (RFP) and for WB, and in numerous publications.

Mouse anti-GFP (Santa Cruz, sc-9996): Manufacturer validated for GFP and GFP mutant fusion proteins and for WB, and in numerous publications.

Mouse anti-Pab1p (Cat# MCA-1G1): Manufacturer validated in Saccharomyces cerevisiae (yeast polyA binding protein (Pab1)) for WB and in numerous publications.

Rabbit anti-FLAG (Cat# F7425): Manufacturer validated in any species (species independent, FLAG epitope recognition) for WB and in numerous publications.
